# Nanotechnology as a Tool to Mitigate the Effects of Intestinal Microbiota on Metabolization of Anthocyanins

**DOI:** 10.3390/antiox11030506

**Published:** 2022-03-05

**Authors:** Thiécla Katiane Osvaldt Rosales, Neuza Mariko Aymoto Hassimotto, Franco Maria Lajolo, João Paulo Fabi

**Affiliations:** 1 Department of Food Science and Experimental Nutrition, School of Pharmaceutical Sciences, University of São Paulo, São Paulo 05508000, Brazil; thieclarosales@usp.br (T.K.O.R.); aymoto@usp.br (N.M.A.H.); fmlajolo@usp.br (F.M.L.); 2 Food and Nutrition Research Center (NAPAN), University of São Paulo, São Paulo 05508080, Brazil; 3 Food Research Center (FoRC), CEPID-FAPESP (Research, Innovation and Dissemination Centers, São Paulo Research Foundation), São Paulo 05508080, Brazil

**Keywords:** anthocyanins, antioxidant activity, biotransformation, controlled delivery, intestinal bacteria, metabolism, nanoencapsulation, phenolic compounds, oxidative stress, polysaccharide-based, protein-based, lipid-based

## Abstract

Anthocyanins are an important group of phenolic compounds responsible for pigmentation in several plants. For humans, a regular intake is associated with a reduced risk of several diseases. However, molecular instability reduces the absorption and bioavailability of these compounds. Anthocyanins are degraded by external factors such as the presence of light, oxygen, temperature, and changes in pH ranges. In addition, the digestion process contributes to chemical degradation, mainly through the action of intestinal microbiota. The intestinal microbiota has a fundamental role in the biotransformation and metabolization of several dietary compounds, thus modifying the chemical structure, including anthocyanins. This biotransformation leads to low absorption of intact anthocyanins, and consequently, low bioavailability of these antioxidant compounds. Several studies have been conducted to seek alternatives to improve stability and protect against intestinal microbiota degradation. This comprehensive review aims to discuss the existing knowledge about the structure of anthocyanins while discussing human absorption, distribution, metabolism, and bioavailability after the oral consumption of anthocyanins. This review will highlight the use of nanotechnology systems to overcome anthocyanin biotransformation by the intestinal microbiota, pointing out the safety and effectiveness of nanostructures to maintain molecular stability.

## 1. Introduction

In recent years, nanotechnology has been considered an important tool for the smart delivery of bioactive compounds in the human body. Nanoencapsulation can be an alternative for the accurate release of phenolic compounds, such as anthocyanins, in the human intestine, thus preserving some biological beneficial effects. Nanoencapsulated anthocyanins can be protected from several factors related to human digestion, mainly biotransformation caused by intestinal microbiota while improving absorption [1,2].

Anthocyanins are of great interest because of their many biological activities. Anthocyanins are soluble vegetable pigments from the class of flavonoids [3,4]. The strict connection between anthocyanin and intestinal microbiota has been studied for many years. Regular consumption of anthocyanins can promote intestinal homeostasis, stimulating the growth of beneficial bacteria, thus improving human health [5,6]. On the other hand, intestinal bacteria have a fundamental role in the metabolization of anthocyanins, leading to structural degradation and biotransformation [5,7,8] and also to the production of bioactive metabolites in a reciprocal interaction [9]. The change in anthocyanin molecular structure reduces the absorption and the possible beneficial effects of intact molecules [9,10], but anthocyanins’ metabolites can also be beneficial to humans.

To minimize the extensive degradation of the aromatic ring structures of anthocyanins by microbiota and protect from other factors responsible for biotransformation in the gastrointestinal tract, researchers have been developed viable alternatives to overcome this massive loss [11,12,13]. Polysaccharides, proteins, and lipids are indicated as potential nanocarriers for anthocyanin-loaded systems [14,15,16,17]. Nanoencapsulated anthocyanins can be protected and have controlled release, increasing the absorption of compounds in their integral form with an improvement in bioavailability and antioxidant activity in specific target tissues [18,19,20]. Therefore, this comprehensive review provides knowledge about the role of intestinal microbiota in extensive metabolization and the relation with other diverse health benefits of anthocyanins. Furthermore, the review contains a wide discussion on the possible use of nanotechnology to minimize the effects of microbiota action on anthocyanins and to improve controlled intestinal delivery.

## 2. Anthocyanins and Human Health: Regular Consumption and Associated Benefits

Epidemiological, clinical, and nutritional studies support the evidence of the relationship between the intake of determined classes of food and human health. Studies point to ingestion benefits of fruits and vegetables, since the consumption of these classes of foods has been associated with a reduction in the risk of developing noncommunicable diseases [20,21,22]. In addition, the benefits for human health are related to the ingestion of polyphenolic compounds, such as anthocyanins, as well as some other plant-derived compounds [23,24,25,26,27].

Anthocyanins are water-soluble compounds that are responsible for pigmentation in several plants. Anthocyanins are one of the major subclasses of flavonoids, a class of polyphenols [28]. These phenolic compounds are derived from secondary plant metabolism, mainly distributed in the vacuoles that are inside cell walls (leaves, flowers, and fruits) providing a wide spectrum of colors, such as blue, red, and purple [29]. The color spectrum is directly affected by changes in pH. In acidic conditions anthocyanins have a red color, and when the pH increases, they turn into blue color. Food sources with high anthocyanin content are blackberries, blueberries, strawberries, grapes, and some tropical fruits [30].

It is widely described in the literature that the consumption of anthocyanin-rich foods is associated with various positive effects on human health [6,20,31]. The functional dietary properties are associated with the inhibition of oxidative stress due to potent antioxidant activity and some other metabolic regulations. Thus, when these compounds are ingested regularly, they can contribute to a reduction in the risk of several diseases whose genesis is oxidative stress with further metabolic impairments [24,27].

The main biological effect observed for anthocyanins is the effective antioxidant capacity [3,28,29,30]. They can easily donate protons to highly reactive free radicals, preventing propagation and further radical formation. These compounds are considered excellent antioxidants due to several characteristics. They have a positive charge, aromatic hydroxyl groups in ideal numbers and organization, a fair degree of structural conjugation, and the presence of electron-donor and electron-withdrawn substituents in the ring structure. All these features break the cycle of the generation of new radicals due to electron deficiency [32,33,34,35]. The main mechanisms involved in the biological activity of anthocyanins are related to the free-radical-neutralization pathway, the cyclooxygenase pathway, the protein-kinase pathway, and the signaling of inflammatory cytokines. Anthocyanins can interrupt lipid-oxidation reactions through radical scavenging or as metal chelators to convert metal hydroperoxides or pro-oxidants to stable compounds [32,33]. The outstanding antioxidant capacity of anthocyanins is observed in several in vitro studies. Anthocyanins can neutralize free radicals by donating an electron or hydrogen atom to an extensive range of reactive oxygen species (ROS), such as superoxide (O_2_^−^), singlet oxygen (^1^O_2_), peroxide (RCOO^•^), hydrogen peroxide (H_2_O_2_), hydroxyl radical (OH·), hypochlorous acid (HOCl^−^), peroxynitric acid (ONOOH^−^), and reactive nitrogen species in a terminator reaction [36,37,38,39,40].

The neutralization of radicals by anthocyanins protects cells from oxidative damage, decreasing the risks of aging and various diseases. In this context, many in vitro and in vivo studies confirmed the health benefits attributed to anthocyanins, such as their antioxidant role [30,37,38,39,40,41,42,43,44], anti-inflammatory action [45], neuroprotection [46,47,48,49], anticancer effects [20,46,47,48,49,50,51,52,53,54,55,56], antiobesity effects [21,57,58,59,60,61,62,63,64], cardiovascular protection [65,66,67], antidiabetic effects [68,69,70,71,72,73], visual protection [74,75,76], and antimicrobial properties [72,73,77,78]. A recent systematic review of 44 randomized controlled trials and 15 prospective studies relating to cardiovascular diseases and ingestion of anthocyanin-rich foods or pure anthocyanins showed strong evidence of their effect on improving the blood lipid profile and decreasing circulating proinflammatory cytokines, justifying their inclusion in a cardioprotective diet [73].

Particularly important is the potential effect of anthocyanins on brain health. They have shown neuro-anti-inflammatory properties and promising protection against neurodegeneration diseases associated with aging [79]. In this respect, particularly challenging is the passage of the blood–brain barrier and delivering these compounds to the brain. Some anthocyanins can cross the barrier while others cannot, and in this case, their effect is not in loco but indirect and due to improvement of local circulation [80].

Recently, the antioxidant and anti-inflammatory activities of anthocyanins from *Lycium ruthenicum* Murray were evaluated in animal models after long-term ingestion. The analyzed results indicate that the antioxidant status in the liver was increased and the inflammatory status in the colon was decreased, with a beneficial modulation of intestinal microbiota. Moreover, researchers noticed an increase in short-chain fatty acids in the cecal content and feces. These results are important to prove the long-term effects of anthocyanin intake and support the idea that enriching foods with anthocyanins is effective in modulating intestinal microbiota [24]. The modulation of microbiota is especially relevant to aging [81].

Another important application of anthocyanins is in the food industry. Due to its coloring capacity and water solubility, it allows for easy incorporation into aqueous food systems [82]. Anthocyanin-rich extracts are becoming increasingly attractive for use as a natural substitute for synthetic dyes in the food and pharmaceutical industries, which is an excellent ecologically sustainable alternative [38]. The use of anthocyanins can benefit the sensory quality of food products. Furthermore, the outstanding antioxidant capacity (decrease lipid and protein oxidation) of these flavonoids stimulates several approaches to enable wide technological applicability in the food industry [83,84,85,86,87]. Despite the relationship between health and anthocyanin consumption being evident, and their use in the food industry increasing as natural colorants or even as dietary supplements, the biological characteristics of anthocyanins are directly related to the preservation of molecular stability [33,77].

## 3. Anthocyanin: Chemical Structure and Molecular Stability

Structurally, anthocyanins are in glycosylated form, the basic structure is constituted by an anthocyanidin nucleus linked to sugars and organic acids [75,78]. Anthocyanins possess two benzene rings linked by a linear three-carbon chain. Anthocyanins are soluble in polar solvents (methanol, ethanol, and water). Acidified methanol (stabilization of the flavylium cation) is widely used for extraction [87,88,89].

**Figure 1:**
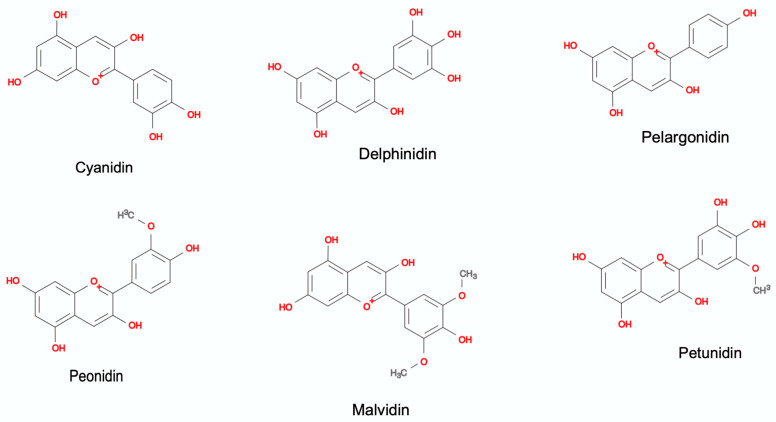
Molecular structure of anthocyanidins (Cyanidin, Delphinidin, Pelargonidin, Peonidin, Malvidin, and Petunidin). The figure was created with Mind the Graph (https://mindthegraph.com (accessed on 10 February 2022)).

More than 635 anthocyanins (six common aglycones and various types of glycosylation and acylation forms) have been identified in nature [29]. Because free anthocyanins are unstable, they are mostly found in glycoside form (galactose, rhamnose, arabinose, xylose, and glucuronic acid are the most common) [89]. In addition, some organic acids can be found attached to the hydroxyl groups on the nucleus and/or to the glycosyl units of anthocyanins [90]. Six major glycoside compounds are found in nature, based on the variation of hydroxylation and methoxylation on aromatic rings and also based on the number and positions of the substituents: pelargonidin, delphinidin, cyanidin, peonidin, petunidin, and malvidin [91]. Cyanidin-3-*O*-glucoside and Malvidin-3-*O*-glucoside are the predominant anthocyanins in plants, especially in fruits. They has a positive charge on the C-ring oxygen atom of the basic flavonoid structure [87]. These compounds have structural variations, such as the position and number of hydroxyl in the molecule, the degree of methylation, and the nature and number of the linked sugar molecule [79,83]. Figure 1 shows the molecular structure of anthocyanidins (aglycone form).

**Figure 2:**
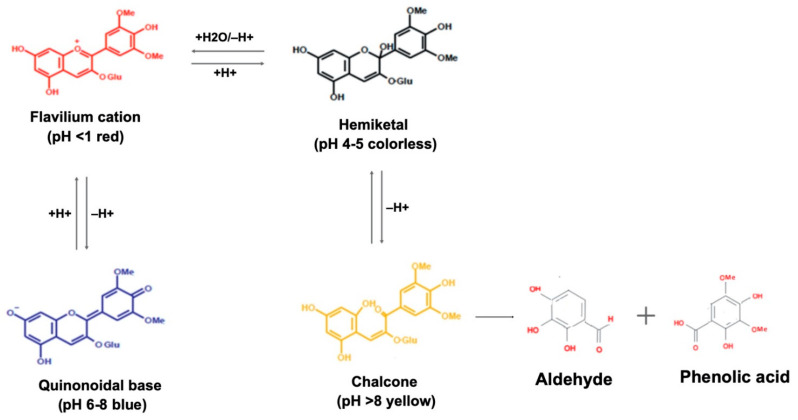
Structural transformation of anthocyanidins at acidic to neutral conditions. The figure was created with Mind the Graph (https://mindthegraph.com (accessed on 10 February 2022)).

However, the color and molecular stability of these pigments are influenced by various factors, such as molecular structure, pH changes, exposure to light, proteins and metallic ions, enzymatic action, and intestinal microbiota [92]. The chemical structure of these compounds, mainly the number and position of the hydroxyl group (-OH) and methoxy groups (-OCH3), influences the molecular stability [25,84]. Furthermore, the pH has a significant influence on the structure and color of the anthocyanins. The variations in pH result in different molecular balances, in which at low pH (acidic) the anthocyanins are particularly red and predominantly in the form of flavylium cation; at slightly acidic pH the structure is colorless hemiketal; and hemiketal chalcone is converted in chalcone by a ring-opening with a yellowish coloration, which at basic pH predominates the quinoidal structure and purple/blue coloration (Figure 2) [93]. The presence of glycosides increases water solubility; on the other hand, acetylation provides higher stability to the anthocyanin molecule [94]. Other factors such as high temperature, processing, storage, and the presence of oxygen also affect stability [95].

Isolated anthocyanins are highly unstable and susceptible to chemical degradation [96]; thus, the measure of human bioavailability and the incorporation into food products are significant challenges [97]. Moreover, molecular instability restricts the use of natural colorants in food systems for processing, formulation, and storage conditions [98]. Thus, due to coloring properties and the numerous health benefits, researchers are involved in exploring the natural potential of anthocyanins. They are interested in developing approaches to maintain molecular structure during food processing and storage through identifying viable alternatives to protect the molecule during digestion, mainly to mitigate the action of the intestinal microbiota.

## 4. Anthocyanin Biotransformation by Human Intestinal Microbiota

In recent decades, research has been directed towards elucidating the complex relationship between anthocyanin consumption and the role of intestinal microbiota. Evidence indicates that long-term consumption of anthocyanins can positively influence human health through positive modulation of intestinal microbiota [23,91]. In addition, microbiota interferes in fundamental biological functions such as absorptive events. The intestinal microbiota is made up of more than a trillion microorganisms established in symbiosis with the host. The systemic effects of the microbiota include immune defense, maintenance of the intestinal barrier, and decreased colonization of potentially pathogenic microorganisms [99,100,101]. Intestinal dysbiosis can impair the bioavailability of numerous essential and nonessential food components. A balanced microbiota provides an increase in intestinal villi and may reduce the risk of developing diseases such as cancer [7,94,95].

Anthocyanins when consumed regularly in foods or supplements can modify the composition of intestinal microbiota, mainly bacteria. In vitro and in vivo studies indicate that certain bacteria with pathogenic potential can have their growth inhibited. On the other hand, the metabolization of anthocyanins by the microbiota can benefit the growth of beneficial bacteria [57,96,97,98]. The main effects of anthocyanins on the intestinal microbiota are related to changes in the composition of bacteria, favoring the specific improvement of the intestinal microbiota population, such as an increase in Bacteroidetes and a decrease in Firmicutes [4]. The imbalance in the bacterial population was observed in animal models induced to obesity (fat diet) but supplemented with high doses of anthocyanins. A reduction in the proportion between the number of Firmicutes and fecal Bacteroidetes was observed, indicating that supplementation with anthocyanins could modulate the animal’s microbiota, thus favoring the reversal of obesity [99,100]. Some factors influence the metabolism of anthocyanins by the intestinal microbiota. The daily ingested dose, the structure of anthocyanin, and interindividual differences have direct interference in the composition of the microbiota [101,102].

The metabolism of anthocyanins by intestinal bacteria involves a sequence of chemical cleavages, initially the glycosidic bonds and then the breaking of the anthocyanidin heterocycle and the degradation to phloroglucinol derivatives and benzoic acids [103,104,105,106,107,108]. Absorption of intact anthocyanins is limited [94], and they are degraded by the action of α-rhamnosidase and β-glycosidase, which are needed to catalyze the reaction, releasing sugar moieties from the anthocyanin structure and transforming it into aglycone form (anthocyanidin) [109,110,111,112,113]. Several intestinal bacteria can metabolize anthocyanins, including *Bifidobacterium* spp. and *Lactobacillus* spp., and the consequent metabolites can stimulate the growth of other specific bacteria, thus providing further modulation of the intestinal microbiota [7,10,113,114,115,116].

Other important factors resulting from the metabolism of anthocyanins by the microbiota are related to short-chain fatty-acid production. Acetate, propionate, and butyrate can serve as a substrate for intestinal epithelial-cell growth (favoring nutrient absorption), can decrease the intestinal pH, and also inhibit the growth of pathogenic bacteria [6,7,106]. Furthermore, anthocyanin supplementation can stimulate an increased number of goblet cells and tight junction proteins and improve villi in the intestine [6].

For the metabolites (low molecular weight) derived from the metabolization of anthocyanins, beneficial effects on the health of the host are attributed [23,107,108], such as the formation of protocatechuic and gallic acids that inhibit the growth of pathogenic bacteria [109,110]. Furthermore, a study using *Wistar* rats supplemented for a long period with high doses of cyanidin-3-*O*-glucoside evaluated the effects on the microbiota after exposure to 3-chloro-1,2-propanediol. The study suggests that anthocyanins contributed to the maintenance of a balanced intestinal microbiota in rats. The evaluated anthocyanin proved to be effective in protecting the intestinal mucosa against damage and in stimulating the growth of beneficial bacteria, restricting intestinal dysbiosis [8]. In this sense, some studies using animal models with oral supplementation of anthocyanins (extracted from different sources and with different concentrations) showed that these compounds influenced the composition of intestinal bacteria in a beneficial way [117,118,119,120,121,122,123]. Thus, the health-promoting effects attributed to anthocyanins are associated with the modulation of the intestinal microbiota [7]. However, despite the several positive effects of anthocyanins described in the literature, there is no consensus on doses and time of ingestion because of intestinal-microbiota variability between humans. This knowledge gap indicates the need for more studies related to the establishment of tolerable upper-intake levels and other dietary guidelines for the consumption and supplementation of anthocyanins [39].

In the elderly, the composition of microbiota changes and may lead to a reduction in concentration and diversity of beneficial bacteria, leading to dysbiosis. Interaction of anthocyanins with microbiota that generates health effects is particularly important for the prevention of diseases in the aging population, with minimal side effects that may occur with drugs [9,81].

### Absorption and Metabolism of Anthocyanins

The metabolism of anthocyanins is a complex process that involves various organs and tissues. In the human host, anthocyanins (from different food sources) undergo successive degradation steps by the action of enzymes and intestinal bacteria, as already described. In addition, the intestinal pH could account for the molecular instability of anthocyanins but could also favor the intestinal biotransformation. Within enterocytes, colonocytes, and in the liver, anthocyanins are metabolized in phase I (less frequently) and phase II [124]. The metabolites generated by the breakdown of the anthocyanin structure and endogenous chemical modifications are excreted via biliary secretion, feces, and urine [118].

**Figure 3:**
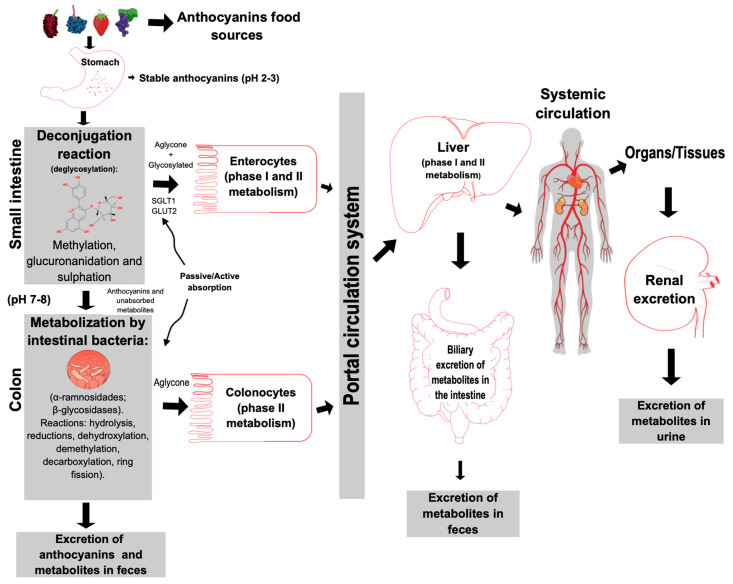
Destination of anthocyanins and their metabolites in the human body after ingestion from food sources. The extensive and successive degradation of anthocyanins by the action of intestinal bacteria and enzymes and the formation of metabolites. After absorption, different organs and tissues are responsible for the metabolization in phases I and II and the excretion of their metabolites. The figure was created with Mind the Graph (https://mindthegraph.com (accessed on 10 February 2022)).

Anthocyanins can cross the stomach (pH 1.5 to 2) in their intact form. In vitro digestion simulation studies have found that anthocyanins are generally stable during incubation with gastric fluids [125,126,127,128,129,130,131,132]. In addition, some studies suggest that there is also absorption in the stomach mucosa, due to the rapid detection of anthocyanin absorption markers in the bloodstream after ingestion of food rich in this compound [104,121,122]. However, most of the absorption occurs in the intestine. In the small intestine (pH 7.4–8), mainly in the jejunum, the absorption of glycosylated forms occurs. Anthocyanidins are passively absorbed after the action of hydrolytic enzymes (changing anthocyanins to the aglycone form) and/or the absorption of glycosylated forms occurs through glucose transporters (SGLT1 and GLUT2) [123,124]. Moreover, the integrity of the intestinal villi is critical for absorption [133]. In enterocytes, anthocyanins undergo phase 2 reactions of metabolism, such as methylation, glucuronidation, and sulfation, catalyzed by UDP-glucuronosyltransferase, sulfotransferases, and catechol-*O*-methyltransferases, respectively [10,125]. Figure 3 illustrates the process of anthocyanin metabolism in the human body from the stomach to the excretion of metabolites.

**Figure 4:**
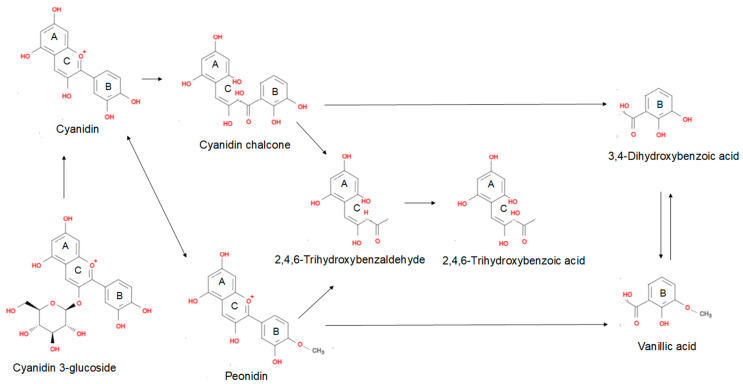
Anthocyanin metabolism by the intestinal microbiota and the formation of different acids. Based on [124,138,139]. The figure was modified from Mind the Graph (https://mindthegraph.com (accessed on 10 February 2022)).

Unabsorbed anthocyanins reach the colon and are metabolized by the colonic microbiota. Most of the absorption of metabolites occurs in the large intestine (pH 7.4–8) [5]. A portion of unabsorbed metabolites and unabsorbed anthocyanins are excreted in feces. A study conducted with patients with ileostomies indicated that most of the anthocyanins arrive in the large intestine intact to be degraded by the microbiota [134]. Additionally, the hydrolysis, reductions, dihydroxylation, demethylation, decarboxylation, and ring fission reactions occur in the colon [127,128]. Bacterial metabolism occurs initially by cleavage of glycosidic bonds, breaking the anthocyanidin heterocycle (C-ring), and degradation to phloroglucinol derivatives (A-ring) and benzoic acids (B-ring) [111]. Figure 4 demonstrates the metabolism of anthocyanins (cyanidin-*O*-glucoside) in the presence of human intestinal bacteria. The degradation process is the result of some conversion steps that are catalyzed by bacterial enzymes in the host. Intestinal bacteria initiate this process by deglycosylation, and then other compounds are formed, such as cyanidin (aglycone), petunidin (a methylation product), and low-molecular-weight catabolites, such as phenolic acids and other phenols. The phenolic acids can then be absorbed by active or passive absorption in the colon and undergo phase 2 enzymatic metabolism [135,136,137].

If absorbed, anthocyanidins and their microbial catabolites are transported through the portal vein and in the liver are distributed to hepatocytes, where they are again metabolized (phases I and II). The products of hepatic metabolism are distributed throughout the tissues and subsequently transported to the enteric system by the bile pathway (an important vehicle for transport) and removed via urinary and/or fecal excretion [35,128].

The absorption of anthocyanins isolated in mixtures or in nanostructured systems is considered a complex mechanism and is not fully elucidated. Anthocyanins may interact differently at diverse absorption sites along the gastrointestinal tract. Advanced techniques are being applied to understand the absorption of these compounds with greater precision, to relate the structure of anthocyanins (isolated or encapsulated) with the absorption and the effect on certain groups of bacteria in the intestinal microbiota. In situ matrix-assisted laser desorption/ionization mass spectrometry imaging can be useful to know the specific sites of absorption and to release anthocyanins (and their metabolites) in different target tissues [138,139,140,141].

The major human metabolites identified in the bloodstream were gallic, vanillic, protocatechuic, 3,4-dihydroxybenzoic, syringic, *p*-cumaric, vanillic, 2,4-dihydroxybenzoic, 2,4,6-trihydroxy benzoic, and 2,4,6-trihydroxy benzoic acids [4,128]. The aglycone form can also be metabolized by intestinal bacteria as a carbon source, decomposing into organic acids such as 3,4-dihydroxyphenylacetic, m-hydroxyphenyl acetic, and *m*-homovanilic acids [131]. However, after ingestion of the anthocyanin source, a limited quantity of intact anthocyanins was detected in the systemic circulation [84,130].

## 5. Biotransformation of Anthocyanins and the Consequent Effect on Bioavailability and Antioxidant Capacity

The metabolism of anthocyanins is complex, and the intense degradation of these compounds limits the bioavailability and the systemic effect. The bioavailability of anthocyanins refers to the amount that are absorbed, reach circulation, suffer metabolization, and are distributed to target tissues [142]. The bioavailability of anthocyanins is very low compared to other flavonoids. In addition to limited absorption and inefficient transport to circulation and distribution, these compounds have high excretion [143]. The biotransformation of anthocyanins by the action of the microbiota leads to less absorption, low biological use, and influences the antioxidant capacity and biological action in specific tissues (39). Inefficient absorption has been reported in some studies, which report that less than 1% of ingested anthocyanins reach the intestine intact and are internalized by enterocytes. Most reports are related to the absorption of metabolites resulting from the degradation of these compounds [102,132,144,145]. Therefore, the low absorption and limited bioavailability of free anthocyanins are due to their susceptibility to high chemical and microbial degradation and excretion rates [133].

The interaction between anthocyanins and the microbiota, and the consequent low bioavailability, has been described in several studies [4,112,113,145,146]. An in vitro study using rat feces evaluated the impact of intestinal bacteria on the degradation of cyanidin-*O*-3-glucoside. The results indicated that anthocyanins were rapidly degraded, which confirms the impact of bacterial action on molecular stability [124]. Some in vivo studies have shown maximum plasma levels of total anthocyanins being 1–100 nM after ingestion of doses at 7–1618 mmol [86,134,135]. After 4 h of ingesting a natural source of anthocyanins, the estimated loss is 60 to 90% that are not detectable in the gastrointestinal tract [94]. In this sense, many in vivo studies have already identified a low absorption and high degradation of anthocyanins by animals and humans [115,147,148,149], probably due to inherent chemical structure but also involving other factors such as food matrix, interaction with nutrients, food processing, and individual factors (genetic and physiological) among other factors [7].

Thus, all mechanisms involved in anthocyanin degradation are still being elucidated. However, the biological activity of the intestinal microbiota is considered an important factor [4,5,6]. Despite the increasing number of studies indicating the possible physiological role of anthocyanin metabolites, greater absorption of anthocyanins (integral form) could increase the antioxidant capacity in specific tissues. In this regard, many researchers are seeking to identify ways to mitigate the effect of microbiota on the biotransformation of anthocyanins [2,11,138,139].

## 6. Nanotechnology Overcoming the Metabolization of Anthocyanins: Biopolymers Delivering Strategies

One of the viable and effective alternatives to minimize the effects of microbiota in the extensive metabolization of anthocyanins is the use of nanotechnology. Nanotechnology is defined as the design, use, and manipulation of materials in systems at the nanometric scale (˂1000 µm) [150,151,152]. Nanocarriers can protect anthocyanin from unfavorable environmental conditions, e.g., pH, temperature, enzyme action, and microbiota degradation [2]. Resistant materials are used to coat the nanostructures, which in addition to protecting anthocyanins during digestion can release them in a controlled manner in the intestine and/or in target cells [11,13]. Furthermore, the anthocyanins encapsulated in the nanostructure could have less interaction with other compounds in the diet, improving bioavailability [2].

A study demonstrated that nanoencapsulated anthocyanin had a greater tolerance to the increase in pH range, the presence of metal ions, and the increase in temperature, thus maintaining the intrinsic capacity of scavenging free radicals [153]. The use of encapsulated anthocyanins, mainly for the formation of biopolyelectrolyte complexes, has shown to maintain stability, overcome chemical degradation, and mitigate the loss of color, thus preserving the bioactivity and enabling their application in foods as natural dyes [154]. The possibility, steps, and strategies were clearly shown in a recent example related to the microencapsulation of polyphenols from *Sambucus nigra* L. [155]. Targeting the intestine is important to control local inflammatory diseases, and recent research designated gut-delivery polyphenols encapsulated with marine polysaccharides as multifunctional nanocarriers [156].

One of the specific chemical properties of anthocyanins refers to their ability to non-covalently interact with some macromolecules to form stable nanostructures [157]. The application of nanocarrier systems for anthocyanin loading can make use of natural polymers, such as polysaccharides, proteins, and lipids [151]. They are pointed out as promising for use as a wall material because they have wide sources of extraction in nature and show excellent biodegradability and biocompatibility [18]. Anthocyanins within the nanostructure are protected from the excessive degradation that happens within the intestinal microbiota. The nanostructures with encapsulated anthocyanins can represent greater absorption of intact molecules by the intestinal mucosa than when free anthocyanins are administered, providing a probable better systemic activity when compared to isolated ingestion [158].

Nanostructures based on polysaccharides can protect and release the encapsulated compounds according to specific physiological stimulation and environment. The physical and chemical properties and functional performance of polysaccharides confer numerous advantages for anthocyanin encapsulation. The complexity of polysaccharide structures is suitable for the construction of nanocarriers. Polysaccharides such as chitosan, cellulose and derivatives, and pectin are widely used for this purpose, protecting and controlling the release of encapsulated bioactive compounds, including anthocyanins [143,144]. Polysaccharide-based nanomaterials are designed for enhancing the responsive delivery that depends on pH, protecting the encapsulated from the intestine environment, and delivering specifically to lower portions of the human intestine. The controlled intestinal release of nanostructures containing anthocyanins can favor absorption, especially in its integral form [153,159,160,161]. The absorption of anthocyanins within polysaccharide nanostructures can occur by recognition of the glycosidic portions of pectin by intestinal epithelial cells in the nanostructures, internalized by the plasma membrane through endosomes, and then the anthocyanins being released in the cell cytoplasm [147,148].

Different types of carbohydrates—natural or modified polysaccharides—are used alone or in combination with other macromolecules to create nanocarriers for anthocyanins delivery [162,163,164,165,166,167,168,169,170,171,172]. Polysaccharide-based nanoencapsulation is suitable for protection, stability, and bioavailability in nanoencapsulation. Over the years, many studies demonstrated the success in the utilization of various polysaccharides for encapsulation of anthocyanins (extract from different sources), such as pectin [164,165,166], chitosan [145,146,152,153,154,155,156,157], and cellulose [173].

The interaction between anthocyanins (cyanidin-3-*O*-glucoside) and citrus pectin with different esterification patterns was investigated using thoroughly explored analytical techniques (isothermal titration calorimetry, nuclear magnetic resonance, and UV-Visible spectrophotometry). The study showed interactions between anthocyanin and pectin, depending on the degree of polysaccharide esterification. It was also reported that a combination of these two compounds had an impact on color maintenance and anthocyanin stability [174]. Furthermore, polysaccharides can form gels when hydrated, have the highest swelling ability, and are ionizable in certain pH ranges, which favors the controlled release and the ability to adhere to animal mucus which improves the delivery to certain organs/tissues [160,161]. In addition, some polysaccharides can be slowly fermented by human intestinal bacteria to an energetic substrate, which will release the encapsulated anthocyanin gradually, thus mitigating molecular degradation that occurs in the intestinal environment [172,175,176,177,178]. Polysaccharides can also interact with proteins forming stable nanostructures at variable pH, therefore protecting encapsulated anthocyanins [162,163].

Proteins are biopolymers extensively used to fabricate nanostructures for the encapsulation of bioactive molecules. Proteins (animal or plant origin) alone or in combination with polysaccharides or other protein molecules can be efficiently used for anthocyanin nanoencapsulation. The protein (natural or denatured form) disposed in the nanostructure provides greater stability to the whole nanocomplex [179]. Examples of proteins used for nanoencapsulation of bioactive compounds are β-Lactoglobulin [1], lysozyme [164], and whey protein [166]. The interaction between anthocyanins at different concentrations (from black rice) and isolated soybean protein was analyzed using three-dimensional fluorescence and Fourier transform infrared spectroscopy. It was observed that anthocyanins are linked by covalent and noncovalent interactions to proteins, with the anthocyanin-protein nanocomplex formed showing a promising use in food formulations [180]. Additionally, the effect of proteins on the stability and bioaccessibility of anthocyanins was recently evaluated. Bovine serum albumin at different concentrations was added to anthocyanins extracted from blueberries in a simulated digestion system and simulated different food processing and storage. The stability and antioxidant capacity of anthocyanins were maintained with the addition of protein, specifically at 0.15 mg/mL. This fact indicated that there was a possible inhibition of anthocyanin degradation by added proteins, thus maintaining the antioxidant capacity [181].

Lipids are also considered suitable as nanocarriers for anthocyanin encapsulation. Lipid-based nanoencapsulation can provide high encapsulation efficiency, controlled release in the intestine, low toxicity, and the excellent possibility of production on an industrial scale. Lipid-based nanoencapsulation can be formed by bilayer structures (usually spherical) with specific polar lipids dispersed in aqueous phases [166,167]. Lipid-based carriers include nanoemulsions, nanoliposomes, solid-lipid nanoparticles, and novel generation of an encapsulation system, namely the nanostructured lipid carrier [182,183,184,185,186]. Some studies using lipids as nanocarriers were successful in maintaining the stability of anthocyanins and preserving the anthocyanin’s chemical structures in a diverse environment [169,170].

In general, biopolymers (polysaccharides, proteins, and lipids) can be applied to the optimization of encapsulation systems. They can be modified or used in their natural form, combined or isolated, and built by different techniques to create smart delivery systems. Biopolymers have potential advantages, such as excellent physicochemical properties, capacity, and functionalities for anthocyanin stabilization techniques [171,172]. However, the nanoencapsulation techniques and the derived nanocompounds should be thoroughly studied by in vitro and in vivo approaches. This is because nanoencapsulated anthocyanins could not perform an ideal antioxidant capacity as the nonencapsulated compounds or could even be degraded if overheated during fabrication; they may even not be released in the target tissue [166]. In this way, researchers must explore the simulated digestion and anthocyanin release in distinct biological systems. Nanoencapsulated anthocyanins may also decrease the effect of food matrices on their absorption [97]. Therefore, incorporating anthocyanins into different food systems is challenging, and nanoencapsulation can be a viable and effective option. It is possible to add them to foods, supplements, and dietetics products [97,187,188]. This could also be a form of increasing the use of underexploited regional fruits and residues from the food industry to develop new products with added economical value, and to explore the existing biodiversity sustainably. Various techniques have been reported in designing nanocarriers based on polysaccharides, proteins, and lipids applied in nanoencapsulation anthocyanins; some of these studies are shown in Table 1. 

In addition, microencapsulation can also be used to stabilize anthocyanins. This encapsulation technology is widely studied to provide greater molecular stability, preserve the antioxidant activity, improve bioaccessibility, and confer controlled-release properties to anthocyanins. Microencapsulation is a process in which the bioactive compound is coated with a specific material to protect against adverse environmental conditions—such as food storage—and intrinsic factors of human digestion [189,190,191,192,193]. In general, microencapsulation refers to the elaboration of a particle with a diameter from 1–1000 µm. There are several types of materials used to microencapsulate anthocyanins, as well as a wide variety of methods for microencapsulation, depending on the purpose of the application, the availability of equipment, and other factors [151,155,189,193].

The methods for elaborating microencapsulated systems can be physical (lyophilization, spray drying, freeze drying, electrospinning/electrospraying), chemical (inclusion complexes), or a combination of both (emulsification, liposomal systems, ionic gelation, and coacervation) [193]. The main biopolymers that can be used as encapsulants are polysaccharides, such as starch, chitosan, pectin, natural gums, mucilage, cellulose, and its derivatives [194,195,196]. Proteins such as whey, caseinate, gelatin, and soy protein are widely used [151,193,197]. The microencapsulation of anthocyanins can be an effective method for the stability, maintenance of color, and antioxidant activity, and has potential for industrial application in foods [151,193,198].

**Table 1:** In vitro studies of nanoencapsulation of anthocyanins (polysaccharides, proteins, and lipid-based) for different purposes.

Source	Nanoencapsulant	Nanoencapsulation Technique	Average Size (nm)	Purpose	Reference
Commercial anthocyanin-rich extract	Whey Protein Isolate and Pectin	Thermal processing and electrostatic complexation	200	Increase antioxidant capacity	[166]
Red cabbage	Palmitic acid and surfactants	Emulsion	455	Stability and antioxidant capacity	[199]
Black rice bran	Chitosan and Alginate	Ionic pre-gelation and polyelectrolyte complex	219.53	Stability	[170]
Blueberry	Carboxymethyl Chitosan	Self-assembly	219.53	Protection and stability	[171]
Açai berry	Eudragit^®^ L100	Modified double-emulsion solvent extraction/evaporation	570–620	Safety	[173]
Blueberry	Chitosan Hydrochloride, Carboxymethyl Chitosan	Electrostatic interaction	178.1	Stability and bioavailability	[169]
Blueberry	Whey Protein, Polyglycerol Polyricinoleate	Nanoemulsion	˂400	Protection and stability	[185]
Natural Source Plant	Lecithin and Cholesterol	Nanoliposomal	53.01	Stability and bioavailability	[186]
Blueberry	Chitosan Hydrochloride, Carboxymethyl Chitosan, and β-Lactoglobulin	Electrostatic interaction	91.71	Stability and bioavailability	[168]
Black rice	Chitosan/Chondroitin sulfate	Self-assembly	350.1	Antioxidant capacity	[200]
Red raspberry pomace	β-Lactoglobulin	Desolvation	129.13–351.85	Stability and bioavailability	[1]
Bilberry	Chitosan and Pectin	Self-assembly	100–300	Stability and bioavailability	[172]
Black carrot	Chitosan	Ionic gelation	274	Increase antioxidant capacity	[201]
Blackberry Commercial anthocyanin-rich extract	Pectin and Lysozyme Casein and Carboxymethyl Cellulose	Self-assembly Self-assembly	198.5 209.9	Protection and stability Stability	[164] [202]

## 7. Conclusions and Future Trends

Anthocyanins have a wide spectrum of biological activities, such as antioxidant, anti-inflammatory, or chemopreventive features, which support human health, although their low bioavailability and extensive biotransformation interfere with these advantages. Studies point to the promising application of nanotechnology tools to encapsulate anthocyanins, thus representing a beneficial alternative to maintain molecular stability. Although the studies were successful in the nanoencapsulation of anthocyanins, in vivo studies (animal and human) are still an unexplored field of research. Several studies indicate the promising application of nanoencapsulation anthocyanins in foods, favoring stability during food processing and storage, preservation of sensory characteristics, resistance to environmental conditions, and digestion factors. Future research could focus on the development of fortified foods and nutritional supplements with nanoencapsulated anthocyanins, increasing the supply of food products beneficial to human health. Although all evidence supports the biological beneficial effects of anthocyanin nanoencapsulation, further studies are needed to determine values limits for safe intake. Natural biopolymers demonstrated adequate biocompatibility, biodegradability, and efficiency for anthocyanin delivery and increased bioavailability. Nanoencapsulation based on polysaccharides, proteins, and lipids can protect anthocyanins through the gastrointestinal tract, releasing them in a controlled manner. The use of nanotechnology for smart protection, controlled delivery, and tissue-specific delivery can minimize the effects of microbiota on the biotransformation of anthocyanins, which represents more effective absorption of intact forms and preservation of biological effects, such as antioxidant activity and some other metabolic modulations.

## References

[1] Salah M., Mansour M., Zogona D., Xu X. (2020). Nanoencapsulation of anthocyanins-loaded β-lactoglobulin nanoparticles: Characterization, stability, and bioavailability in vitro. Food Res. Int..

[2] Salarbashi D., Bazeli J., Rad E.F. (2020). An update on the new achievements in the nanocapsulation of anthocyanins. Nanomed. J..

[3] Zhou L., Xie M., Yang F., Liu J. (2019). Antioxidant activity of high purity blueberry anthocyanins and the effects on human intestinal microbiota. LWT.

[4] Faria A., Fernandes I., Norberto S., Mateus N., Calhau C. (2014). Interplay between Anthocyanins and Gut Microbiota. J. Agric. Food Chem..

[5] Igwe E.O., Charlton K.E., Probst Y.C., Kent K., Netzel M.E. (2018). A systematic literature review of the effect of anthocyanins on gut microbiota populations. J. Hum. Nutr. Diet..

[6] Verediano T., Martino H.S.D., Paes M.D., Tako E. (2021). Effects of Anthocyanin on Intestinal Health: A Systematic Review. Nutrients.

[7] Tian L., Tan Y., Chen G., Wang G., Sun J., Ou S., Chen W., Bai W. (2019). Metabolism of anthocyanins and consequent effects on the gut microbiota. Crit. Rev. Food Sci. Nutr..

[8] Chen G., Wang G., Zhu C., Jiang X., Sun J., Tian L., Bai W. (2019). Effects of cyanidin-3-O-glucoside on 3-chloro-1,2-propanediol induced intestinal microbiota dysbiosis in rats. Food Chem. Toxicol..

[9] Corrêa T.A.F., Rogero M.M., Hassimotto N.M.A., Lajolo F.M. (2019). The Two-Way Polyphenols-Microbiota Interactions and Their Effects on Obesity and Related Metabolic Diseases. Front. Nutr..

[10] Wang D., Xia M., Yan X., Li D., Wang L., Xu Y., Jin T., Ling W. (2012). Gut microbiota metabolism of anthocyanin promotes reverse cholesterol transport in mice via repressing miRNA-10b. Circ. Res..

[11] Fernandes I., Faria A., Calhau C., de Freitas V., Mateus N. (2014). Bioavailability of anthocyanins and derivatives. J. Funct. Foods.

[12] Jafari S.M., McClements D.J. (2017). Nanotechnology Approaches for Increasing Nutrient Bioavailability. Adv. Food Nutr. Res..

[13] Khalil I., Yehye W.A., Etxeberria A.E., Alhadi A.A., Dezfooli S.M., Julkapli N.B.M., Basirun W.J., Seyfoddin A. (2019). Nanoantioxidants: Recent Trends in Antioxidant Delivery Applications. Antioxidants.

[14] Lu X., Chen J., Guo Z., Zheng Y., Rea M.C., Su H., Zheng X., Zheng B., Miao S. (2019). Using polysaccharides for the enhancement of functionality of foods: A review. Trends Food Sci. Technol..

[15] Akhavan S., Assadpour E., Katouzian I., Jafari S.M. (2018). Lipid nano scale cargos for the protection and delivery of food bioactive ingredients and nutraceuticals. Trends Food Sci. Technol..

[16] Assadpour E., Jafari S.M. (2019). An overview of biopolymer nanostructures for encapsulation of food ingredients. Biopolymer Nanostructures for Food Encapsulation Purposes.

[17] Sadeghi R., Mehryar L., Karimi M., Kokini J. (2017). Nanocapsule formation by individual biopolymer nanoparticles. Nanoencapsulation Technologies for the Food and Nutraceutical Industries.

[18] Ayala-Fuentes J.C., Chavez-Santoscoy R.A. (2021). Nanotechnology as a Key to Enhance the Benefits and Improve the Bioavailability of Flavonoids in the Food Industry. Foods.

[19] Arpagaus C. (2019). Production of food bioactive-loaded nanoparticles by nano spray drying. Nanoencapsulation of Food Ingredients by Specialized Equipment.

[20] Hosseini S.M.H., Ghiasi F., Jahromi M. (2017). Nanocapsule formation by complexation of biopolymers. Nanoencapsulation Technologies for the Food and Nutraceutical Industries.

[21] Li D., Wang P., Luo Y., Zhao M., Chen F. (2017). Health benefits of anthocyanins and molecular mechanisms: Update from recent decade. Crit. Rev. Food Sci. Nutr..

[22] Park E., Edirisinghe I., Wei H., Vijayakumar L.P., Banaszewski K., Cappozzo J.C., Burton-Freeman B. (2016). A dose-response evaluation of freeze-dried strawberries independent of fiber content on metabolic indices in abdominally obese individuals with insulin resistance in a randomized, single-blinded, diet-controlled crossover trial. Mol. Nutr. Food Res..

[23] Overall J., Bonney S.A., Wilson M., Beermann A., Grace M.H., Esposito D., Lila M.A., Komarnytsky S. (2017). Metabolic Effects of Berries with Structurally Diverse Anthocyanins. Int. J. Mol. Sci..

[24] Peng Y., Yan Y., Wan P., Dong W., Huang K., Ran L., Mi J., Lu L., Zeng X., Cao Y. (2020). Effects of long-term intake of anthocyanins from Lycium ruthenicum Murray on the organism health and gut microbiota in vivo. Food Res. Int..

[25] Mattioli R., Francioso A., Mosca L., Silva P. (2020). Anthocyanins: A Comprehensive Review of Their Chemical Properties and Health Effects on Cardiovascular and Neurodegenerative Diseases. Molecules.

[26] Garcia C., Blesso C.N. (2021). Antioxidant properties of anthocyanins and their mechanism of action in atherosclerosis. Free Radic. Biol. Med..

[27] Tena N., Martín J., Asuero A.G. (2020). State of the Art of Anthocyanins: Antioxidant Activity, Sources, Bioavailability, and Therapeutic Effect in Human Health. Antioxidants.

[28] Boroski M., Visentaner J.S., Cottica S.M., Morais D.M. (2015). Antioxidantes: Princípios e Métodos Analíticos.

[29] He J., Giusti M.M. (2010). Anthocyanins: Natural Colorants with Health-Promoting Properties. Annu. Rev. Food Sci. Technol..

[30] Pina F., Oliveira J., Freitas V. (2015). Anthocyanins and derivatives are more than flavylium cations. Tetrahedron.

[31] Denev P., Číž M., Kratchanova M., Blazheva D. (2019). Black chokeberry (*Aronia melanocarpa*) polyphenols reveal different antioxidant, antimicrobial and neutrophil-modulating activities. Food Chem..

[32] Heinonen I.M., Meyer A.A.S., Frankel E.N. (1998). Antioxidant Activity of Berry Phenolics on Human Low-Density Lipoprotein and Liposome Oxidation. J. Agric. Food Chem..

[33] Jennings A., Welch A.A., Fairweather-Tait S.J., Kay C., Minihane A.-M., Chowienczyk P., Jiang B., Cecelja M., Spector T., MacGregor A. (2012). Higher anthocyanin intake is associated with lower arterial stiffness and central blood pressure in women. Am. J. Clin. Nutr..

[34] Wang S.Y., Lin H.-S. (2000). Antioxidant Activity in Fruits and Leaves of Blackberry, Raspberry, and Strawberry Varies with Cultivar and Developmental Stage. J. Agric. Food Chem..

[35] Leopoldini M., Russo N., Toscano M. (2011). The molecular basis of working mechanism of natural polyphenolic antioxidants. Food Chem..

[36] Perron N.R., Brumaghim J.L. (2009). A Review of the Antioxidant Mechanisms of Polyphenol Compounds Related to Iron Binding. Cell Biochem. Biophys..

[37] Zhang H., Tsao R. (2016). Dietary polyphenols, oxidative stress and antioxidant and anti-inflammatory effects. Curr. Opin. Food Sci..

[38] Salehi B., Sharifi-Rad J., Cappellini F., Reiner Ž., Zorzan D., Imran M., Sener B., Kilic M., El-Shazly M., Fahmy N.M. (2020). The Therapeutic Potential of Anthocyanins: Current Approaches Based on Their Molecular Mechanism of Action. Front. Pharmacol..

[39] Smeriglio A., Barreca D., Bellocco E., Trombetta D. (2016). Chemistry, Pharmacology and Health Benefits of Anthocyanins. Phytother. Res..

[40] Magalhaes L.M., Segundo M.A., Reis S., Lima J.L. (2008). Methodological aspects about in vitro evaluation of antioxidant properties. Anal. Chim. Acta.

[41] Hassimotto N.M.A., Genovese M.I., Lajolo F.M. (2009). Antioxidant capacity of Brazilian fruit, vegetables and commercially-frozen fruit pulps. J. Food Compos. Anal..

[42] Zafra-Stone S., Yasmin T., Bagchi M., Chatterjee A., Vinson J.A., Bagchi D. (2007). Berry anthocyanins as novel antioxidants in human health and disease prevention. Mol. Nutr. Food Res..

[43] Wang J.-H., Xu J.-L., Zhang J.-C., Liu Y., Sun H.-J., Zha X. (2015). Physicochemical properties and antioxidant activities of polysaccharide from floral mushroom cultivated in Huangshan Mountain. Carbohydr. Polym..

[44] Stintzing F.C., Stintzing A.S., Carle R., Frei B., Wrolstad R.E. (2002). Color and Antioxidant Properties of Cyanidin-Based Anthocyanin Pigments. J. Agric. Food Chem..

[45] Joseph S.V., Edirisinghe I., Burton-Freeman B.M. (2014). Berries: Anti-Inflammatory effects in humans. J. Agric. Food Chem..

[46] Tarozzi A., Morroni F., Hrelia S., Angeloni C., Marchesi A., Cantelli-Forti G., Hrelia P. (2007). Neuroprotective effects of anthocyanins and their in vivo metabolites in SH-SY5Y cells. Neurosci. Lett..

[47] Di Giacomo C., Acquaviva R., Santangelo R., Sorrenti V., Vanella L., Volti G.L., D’Orazio N., Vanella A., Galvano F. (2012). Effect of Treatment with Cyanidin-3-O-β-D-Glucoside on Rat Ischemic/Reperfusion Brain Damage. Evid.-Based Complement. Altern. Med..

[48] Kim K.T., Nam T.K., Park Y.S., Kim Y.B., Park S.W. (2011). Neuroprotective effect of anthocyanin on experimental traumatic spinal cord injury. J. Korean Neurosurg. Soc..

[49] Chen G., Bower K.A., Xu M., Ding M., Shi X., Ke Z.-J., Luo J. (2009). Cyanidin-3-Glucoside Reverses Ethanol-Induced Inhibition of Neurite Outgrowth: Role of Glycogen Synthase Kinase 3 Beta. Neurotox. Res..

[50] Lin B.-W., Gong C.-C., Song H.-F., Cui Y.-Y. (2016). Effects of anthocyanins on the prevention and treatment of cancer. J. Cereb. Blood Flow Metab..

[51] Silva L.B.A.R., Pinheiro-Castro N., Novaes G.M., Pascoal G.D.F.L., Ong T.P. (2019). Bioactive food compounds, epigenetics and chronic disease prevention: Focus on early-life interventions with polyphenols. Food Res. Int..

[52] Mok J.W., Chang D.-J., Joo C.-K. (2014). Antiapoptotic Effects of Anthocyanin from the Seed Coat of Black Soybean Against Oxidative Damage of Human Lens Epithelial Cell Induced by H_2_O_2_. Curr. Eye Res..

[53] Wang L.-S., Hecht S., Carmella S.G., Yu N., LaRue B., Henry C., McIntyre C., Rocha C., Lechner J.F., Stoner G.D. (2009). Anthocyanins in Black Raspberries Prevent Esophageal Tumors in Rats. Cancer Prev. Res..

[54] Faria A., Pestana D., Teixeira D., de Freitas V., Mateus N., Calhau C. (2010). Blueberry anthocyanins and pyruvic acid adducts: Anticancer properties in breast cancer cell lines. Phytotherapy Res..

[55] Hui C., Bin Y., Xiaoping Y., Long Y., Chunye C., Mantian M., Wenhua L. (2010). Anticancer Activities of an Anthocyanin-Rich Extract From Black Rice Against Breast Cancer Cells In Vitro and In Vivo. Nutr. Cancer.

[56] Chen X.-Y., Zhou J., Luo L.-P., Han B., Li F., Chen J.-Y., Zhu Y.-F., Chen W., Yu X.-P. (2015). Black Rice Anthocyanins Suppress Metastasis of Breast Cancer Cells by Targeting RAS/RAF/MAPK Pathway. BioMed Res. Int..

[57] Lala G., Malik M., Zhao C., He J., Kwon Y., Giusti M.M., Magnuson B.A. (2006). Anthocyanin-Rich Extracts Inhibit Multiple Biomarkers of Colon Cancer in Rats. Nutr. Cancer.

[58] Lim S., Xu J., Kim J., Chen T.-Y., Su X., Standard J., Carey E., Griffin J., Herndon B., Katz B. (2013). Role of anthocyanin-enriched purple-fleshed sweet potato p40 in colorectal cancer prevention. Mol. Nutr. Food Res..

[59] Jang H., Ha U.-S., Kim S.-J., Yoon B.-I., Han D.-S., Yuk S.-M., Kim S.-W. (2010). Anthocyanin Extracted from Black Soybean Reduces Prostate Weight and Promotes Apoptosis in the Prostatic Hyperplasia-Induced Rat Model. J. Agric. Food Chem..

[60] Bontempo P., De Masi L., Carafa V., Rigano D., Scisciola L., Iside C., Grassi R., Molinari A.M., Aversano R., Nebbioso A. (2015). Anticancer activities of anthocyanin extract from genotyped *Solanum tuberosum* L. “Vitelotte”. J. Funct. Foods.

[61] Jayarathne S., Stull A.J., Park O., Kim J.H., Thompson L., Moustaid-Moussa N. (2019). Protective Effects of Anthocyanins in Obesity-Associated Inflammation and Changes in Gut Microbiome. Mol. Nutr. Food Res..

[62] Esposito D., Damsud T., Wilson M., Grace M.H., Strauch R., Li X., Lila M.A., Komarnytsky S. (2015). Black Currant Anthocyanins Attenuate Weight Gain and Improve Glucose Metabolism in Diet-Induced Obese Mice with Intact, but Not Disrupted, Gut Microbiome. J. Agric. Food Chem..

[63] Badshah H., Ullah I., Kim S.E., Kim T.-H., Lee H.Y., Kim M.O. (2013). Anthocyanins attenuate body weight gain via modulating neuropeptide Y and GABAB1 receptor in rats hypothalamus. Neuropeptides.

[64] Wu T., Tang Q., Yu Z., Gao Z., Hu H., Chen W., Zheng X., Yu T. (2014). Inhibitory effects of sweet cherry anthocyanins on the obesity development in C57BL/6 mice. Int. J. Food Sci. Nutr..

[65] Curtis P.J., Van Der Velpen V., Berends L., Jennings A., Feelisch M., Umpleby A.M., Evans M., Fernandez B.O., Meiss M.S., Minnion M. (2019). Blueberries improve biomarkers of cardiometabolic function in participants with metabolic syndrome—results from a 6-month, double-blind, randomized controlled trial. Am. J. Clin. Nutr..

[66] Rechner A.R., Kroner C. (2005). Anthocyanins and colonic metabolites of dietary polyphenols inhibit platelet function. Thromb. Res..

[67] Toufektsian M.-C., De Lorgeril M., Nagy N., Salen P., Donati M.B., Giordano L., Mock H.-P., Peterek S., Matros A., Petroni K. (2008). Chronic Dietary Intake of Plant-Derived Anthocyanins Protects the Rat Heart against Ischemia-Reperfusion Injury. J. Nutr..

[68] Takikawa M., Inoue S., Horio F., Tsuda T. (2010). Dietary Anthocyanin-Rich Bilberry Extract Ameliorates Hyperglycemia and Insulin Sensitivity via Activation of AMP-Activated Protein Kinase in Diabetic Mice. J. Nutr..

[69] Li D., Zhang Y., Liu Y., Sun R., Xia M. (2015). Purified Anthocyanin Supplementation Reduces Dyslipidemia, Enhances Antioxidant Capacity, and Prevents Insulin Resistance in Diabetic Patients. J. Nutr..

[70] Kang M.-K., Lim S.S., Lee J.-Y., Yeo K.M., Kang Y.-H. (2013). Anthocyanin-Rich Purple Corn Extract Inhibit Diabetes-Associated Glomerular Angiogenesis. PLoS ONE.

[71] Liu Y., Li D., Zhang Y., Sun R., Xia M. (2014). Anthocyanin increases adiponectin secretion and protects against diabetes-related endothelial dysfunction. Am. J. Physiol. Endocrinol. Metab..

[72] Tsuda T., Ueno Y., Aoki H., Koda T., Horio F., Takahashi N., Kawada T., Osawa T. (2004). Anthocyanin enhances adipocytokine secretion and adipocyte-specific gene expression in isolated rat adipocytes. Biochem. Biophys. Res. Commun..

[73] Xu L., Tian Z., Chen H., Zhao Y., Yang Y. (2021). Anthocyanins, Anthocyanin-Rich Berries, and Cardiovascular Risks: Systematic Review and Meta-Analysis of 44 Randomized Controlled Trials and 15 Prospective Cohort Studies. Front. Nutr..

[74] Miyake S., Takahashi N., Sasaki M., Kobayashi S., Tsubota K., Ozawa Y. (2011). Vision preservation during retinal inflammation by anthocyanin-rich bilberry extract: Cellular and molecular mechanism. Lab. Investig..

[75] Paik S.-S., Jeong E., Jung S.W., Ha T.J., Kang S., Sim S., Jeon J.H., Chun M.-H., Kim I.-B. (2012). Anthocyanins from the seed coat of black soybean reduce retinal degeneration induced by N-methyl-N-nitrosourea. Exp. Eye Res..

[76] Ohguro H., Ohguro I., Katai M., Tanaka S. (2012). Two-Year Randomized, Placebo-Controlled Study of Black Currant Anthocyanins on Visual Field in Glaucoma. Ophthalmologica.

[77] Côté J., Caillet S., Doyon G., Dussault D., Sylvain J.-F., Lacroix M. (2011). Antimicrobial effect of cranberry juice and extracts. Food Control.

[78] Puupponen-Pimia R., Nohynek L., Meier C., Kahkonen M., Heinonen M., Hopia A., Oksman-Caldentey K.-M. (2001). Antimicrobial properties of phenolic compounds from berries. J. Appl. Microbiol..

[79] Henriques J.F., Serra D., Dinis T.C.P., Almeida L.M. (2020). The Anti-Neuroinflammatory Role of Anthocyanins and Their Metabolites for the Prevention and Treatment of Brain Disorders. Int. J. Mol. Sci..

[80] Shimazu R., Anada M., Miyaguchi A., Nomi Y., Matsumoto H. (2021). Evaluation of Blood–Brain Barrier Permeability of Polyphenols, Anthocyanins, and Their Metabolites. J. Agric. Food Chem..

[81] Hair R., Sakaki J., Chun O. (2021). Anthocyanins, Microbiome and Health Benefits in Aging. Molecules.

[82] Bridle P., Timberlake C. (1997). Anthocyanins as natural food colours—selected aspects. Food Chem..

[83] Santos-Buelga C., Mateus N., De Freitas V. (2014). Anthocyanins. Plant Pigments and Beyond. J. Agric. Food Chem..

[84] Dias S., Castanheira E.M.S., Gil Fortes A., Pereira D.M., Gonçalves M.S.T. (2020). Natural Pigments of Anthocyanin and Betalain for Coloring Soy-Based Yogurt Alternative. Foods.

[85] Jokioja J., Yang B., Linderborg K.M. (2021). Acylated anthocyanins: A review on their bioavailability and effects on postprandial carbohydrate metabolism and inflammation. Compr. Rev. Food Sci. Food Saf..

[86] Tan C., Celli G.B., Selig M.J., Abbaspourrad A. (2018). Catechin modulates the copigmentation and encapsulation of anthocyanins in polyelectrolyte complexes (PECs) for natural colorant stabilization. Food Chem..

[87] Khoo H.E., Azlan A., Tang S.T., Lim S.M. (2017). Anthocyanidins and anthocyanins: Colored pigments as food, pharmaceutical ingredients, and the potential health benefits. Food Nutr. Res..

[88] Celli G.B., Tan C., Selig M.J. (2018). Anthocyanidins and anthocyanins. Encyclopedia of Food Chemistry.

[89] Pereira D.M., Valentão P., Pereira J.A., Andrade P.B. (2009). Phenolics: From Chemistry to Biology. Molecules.

[90] Rein M.J. (2005). Copigmentation Reactions and Color Stability of Berry. Ph.D. Thesis.

[91] Bueno J.M., Sáez-Plaza P., Ramos-Escudero F., Jiménez A.M., Fett R., Asuero A.G. (2012). Analysis and Antioxidant Capacity of Anthocyanin Pigments. Part II: Chemical Structure, Color, and Intake of Anthocyanins. Crit. Rev. Anal. Chem..

[92] Fleschhut J., Kratzer F., Rechkemmer G., Kulling S.E. (2006). Stability and biotransformation of various dietary anthocyanins in vitro. Eur. J. Nutr..

[93] Brouillard R., Markakis P. (1982). Chemical structure of anthocyanins. Anthocyanins as Food Colors.

[94] Prior R.L., Wu X. (2006). Anthocyanins: Structural characteristics that result in unique metabolic patterns and biological activities. Free Radic. Res..

[95] Cavalcanti R.N., Santos D.T., Meireles M.A.A. (2011). Non-thermal stabilization mechanisms of anthocyanins in model and food systems—An overview. Food Res. Int..

[96] Kamonpatana K., Failla M.L., Kumar P.S., Giusti M.M. (2014). Anthocyanin Structure Determines Susceptibility to Microbial Degradation and Bioavailability to the Buccal Mucosa. J. Agric. Food Chem..

[97] Yousuf B., Gul K., Wani A.A., Singh P. (2016). Health Benefits of Anthocyanins and Their Encapsulation for Potential Use in Food Systems: A Review. Crit. Rev. Food Sci. Nutr..

[98] Giusti M.M., Wrolstad R.E. (2003). Acylated anthocyanins from edible sources and their applications in food systems. Biochem. Eng. J..

[99] Guo J., Yin M., Han X., You Y., Huang W., Zhan J. (2020). The influence of oxygen on the metabolites of phenolic blueberry extract and the mouse microflora during in vitro fermentation. Food Res. Int..

[100] Fernández J., Redondo-Blanco S., Miguélez E.M., Villar C.J., Clemente A., Lombó F. (2015). Healthy effects of prebiotics and their metabolites against intestinal diseases and colorectal cancer. AIMS Microbiol..

[101] Hidalgo M., Concha M.J.O., Kolida S., Walton G.E., Kallithraka S., Spencer J.P.E., Gibson G.R., De Pascual-Teresa S. (2012). Metabolism of Anthocyanins by Human Gut Microflora and Their Influence on Gut Bacterial Growth. J. Agric. Food Chem..

[102] Bischoff S.C. (2011). ‘Gut health’: A new objective in medicine?. BMC Med..

[103] Kataoka K. (2016). The intestinal microbiota and its role in human health and disease. J. Med. Investig..

[104] Stoupi S., Williamson G., Drynan J.W., Barron D., Clifford M.N. (2009). A comparison of the in vitro biotransformation of (-)-epicatechin and procyanidin B2 by human faecal microbiota. Mol. Nutr. Food Res..

[105] Lee H.C., Jenner A.M., Low C.S., Lee Y.K. (2006). Effect of tea phenolics and their aromatic fecal bacterial metabolites on intestinal microbiota. Res. Microbiol..

[106] Jamar G., Estadella D., Pisani L.P. (2017). Contribution of anthocyanin-rich foods in obesity control through gut microbiota interactions. BioFactors.

[107] Luo Y., Fang J.-L., Yuan K., Jin S.-H., Guo Y. (2019). Ameliorative effect of purified anthocyanin from Lycium ruthenicum on atherosclerosis in rats through synergistic modulation of the gut microbiota and NF-κB/SREBP-2 pathways. J. Funct. Foods.

[108] Wang H., Liu D., Ji Y., Liu Y., Xu L., Guo Y. (2020). Dietary Supplementation of Black Rice Anthocyanin Extract Regulates Cholesterol Metabolism and Improves Gut Microbiota Dysbiosis in C57BL/6J Mice Fed a High-Fat and Cholesterol Diet. Mol. Nutr. Food Res..

[109] Van Dorsten F.A., Grün C.H., Van Velzen E.J.J., Jacobs D.M., Draijer R., van Duynhoven J. (2009). The metabolic fate of red wine and grape juice polyphenols in humans assessed by metabolomics. Mol. Nutr. Food Res..

[110] Bolca S., Urpi-Sarda M., Blondeel P., Roche N., Vanhaecke L., Possemiers S., Al-Maharik N., Botting N., De Keukeleire D., Bracke M. (2010). Disposition of soy isoflavones in normal human breast tissue. Am. J. Clin. Nutr..

[111] Aura A.-M., Martin-Lopez P., O’Leary K.A., Williamson G., Oksman-Caldentey K.-M., Poutanen K., Santos-Buelga C. (2005). In vitro metabolism of anthocyanins by human gut microflora. Z. Ernährungswissenschaft.

[112] Fernandes I., Faria A., de Freitas V., Calhau C., Mateus N. (2015). Multiple-approach studies to assess anthocyanin bioavailability. Phytochem. Rev..

[113] Morais C.A., de Rosso V.V., Estadella D., Pisani L.P. (2016). Anthocyanins as inflammatory modulators and the role of the gut microbiota. J. Nutr. Biochem..

[114] Xiao S., Jiang S., Qian D., Duan J. (2019). Modulation of microbially derived short-chain fatty acids on intestinal homeostasis, metabolism, and neuropsychiatric disorder. Appl. Microbiol. Biotechnol..

[115] Cardona F., Andrés-Lacueva C., Tulipani S., Tinahones F.J., Queipo-Ortuño M.I. (2013). Benefits of polyphenols on gut microbiota and implications in human health. J. Nutr. Biochem..

[116] Kutschera M., Engst W., Blaut M., Braune A. (2011). Isolation of catechin-converting human intestinal bacteria. J. Appl. Microbiol..

[117] Ajiboye T.O., Habibu R.S., Saidu K., Haliru F.Z., Ajiboye H.O., Aliyu N.O., Ibitoye O.B., Uwazie J.N., Muritala H.F., Bello S.A. (2017). Involvement of oxidative stress in protocatechuic acid-mediated bacterial lethality. Microbiologyopen.

[118] Kuntz S., Asseburg H., Dold S., Römpp A., Fröhling B., Kunz C., Rudloff S. (2015). Inhibition of low-grade inflammation by anthocyanins from grape extract in an in vitro epithelial-endothelial co-culture model. Food Funct..

[119] Rodríguez-Daza M.C., Daoust L., Boutkrabt L., Pilon G., Varin T., Dudonné S., Levy É., Marette A., Roy D., Desjardins Y. (2020). Wild blueberry proanthocyanidins shape distinct gut microbiota profile and influence glucose homeostasis and intestinal phenotypes in high-fat high-sucrose fed mice. Sci. Rep..

[120] Espley R.V., Butts C.A., Laing W.A., Martell S., Smith H., McGhie T.K., Zhang J., Paturi G., Hedderley D., Bovy A. (2014). Dietary Flavonoids from Modified Apple Reduce Inflammation Markers and Modulate Gut Microbiota in Mice. J. Nutr..

[121] Cao L., Gil Lee S., Melough M.M., Sakaki J.R., Maas K.R., Koo S.I., Chun O.K. (2020). Long-Term Blackcurrant Supplementation Modified Gut Microbiome Profiles in Mice in an Age-Dependent Manner: An Exploratory Study. Nutrients.

[122] Cremonini E., Daveri E., Mastaloudis A., Adamo A.M., Mills D., Kalanetra K., Hester S.N., Wood S.M., Fraga C.G., Oteiza P.I. (2019). Anthocyanins protect the gastrointestinal tract from high fat diet-induced alterations in redox signaling, barrier integrity and dysbiosis. Redox Biol..

[123] Żary-Sikorska E., Fotschki B., Fotschki J., Wiczkowski W., Juśkiewicz J. (2019). Preparations from purple carrots containing anthocyanins improved intestine microbial activity, serum lipid profile and antioxidant status in rats. J. Funct. Foods.

[124] Hanske L., Engst W., Loh G., Sczesny S., Blaut M., Braune A. (2013). Contribution of gut bacteria to the metabolism of cyanidin 3-glucoside in human microbiota-associated rats. Br. J. Nutr..

[125] He J., Wallace T.C., Keatley K.E., Failla M.L., Giusti M.M. (2009). Stability of Black Raspberry Anthocyanins in the Digestive Tract Lumen and Transport Efficiency into Gastric and Small Intestinal Tissues in the Rat. J. Agric. Food Chem..

[126] Pérez-Vicente A., Gil-Izquierdo A., García-Viguera C. (2002). In Vitro Gastrointestinal Digestion Study of Pomegranate Juice Phenolic Compounds, Anthocyanins, and Vitamin C. J. Agric. Food Chem..

[127] McDougall G., Fyffe S., Dobson P., Stewart D. (2005). Anthocyanins from red wine—Their stability under simulated gastrointestinal digestion. Phytochemistry.

[128] Bermúdez-Soto M.J., Tomas-Barberan F.-A., García-Conesa M.T. (2007). Stability of polyphenols in chokeberry (Aronia melanocarpa) subjected to in vitro gastric and pancreatic digestion. Food Chem..

[129] Felgines C., Texier O., Besson C., Fraisse D., Lamaison J.-L., Rémésy C. (2002). Blackberry Anthocyanins Are Slightly Bioavailable in Rats. J. Nutr..

[130] Talavéra S., Felgines C., Texier O., Besson C., Manach C., Lamaison J.-L., Rémésy C. (2004). Anthocyanins Are Efficiently Absorbed from the Small Intestine in Rats. J. Nutr..

[131] Kamiloglu S., Capanoglu E., Grootaert C., Van Camp J. (2015). Anthocyanin Absorption and Metabolism by Human Intestinal Caco-2 Cells—A Review. Int. J. Mol. Sci..

[132] Manach C., Williamson G., Morand C., Scalbert A., Rémésy C. (2005). Bioavailability and bioefficacy of polyphenols in humans. I. Review of 97 bioavailability studies. Am. J. Clin. Nutr..

[133] González-Barrio R., Borges G., Mullen W., Crozier A. (2010). Bioavailability of Anthocyanins and Ellagitannins Following Consumption of Raspberries by Healthy Humans and Subjects with an Ileostomy. J. Agric. Food Chem..

[134] Kahle K., Kraus M., Scheppach W., Ackermann M., Ridder F., Richling E. (2006). Studies on apple and blueberry fruit constituents: Do the polyphenols reach the colon after ingestion?. Mol. Nutr. Food Res..

[135] Braga A.R.C., Murador D.C., de Souza Mesquita L.M., de Rosso V.V. (2018). Bioavailability of anthocyanins: Gaps in knowledge, challenges and future research. J. Food Compos. Anal..

[136] Fang J. (2014). Bioavailability of anthocyanins. Drug Metab. Rev..

[137] Ichiyanagi T., Shida Y., Rahman M.M., Hatano A.Y., Konishi T. (2006). Bioavailability and Tissue Distribution of Anthocyanins in Bilberry (*Vaccinium myrtillus* L.) Extract in Rats. J. Agric. Food Chem..

[138] Chen Y., Li Q., Zhao T., Zhang Z., Mao G., Feng W., Wu X., Yang L. (2017). Biotransformation and metabolism of three mulberry anthocyanin monomers by rat gut microflora. Food Chem..

[139] Zhu Y., Sun H., He S., Lou Q., Yu M., Tang M., Tu L. (2018). Metabolism and prebiotics activity of anthocyanins from black rice (*Oryza sativa* L.) in vitro. PLoS ONE.

[140] Hahm T.H., Tanaka M., Matsui T. (2022). Current Knowledge on Intestinal Absorption of Anthocyanins. J. Agric. Food Chem..

[141] Zou T.B., Feng D., Song G., Li H.W., Tang H.W., Ling W.H. (2014). The role of sodium-dependent glucose transporter 1 and glucose transporter 2 in the absorption of cyanidin-3-O-β-glucoside in caco-2 cells. Nutrients.

[142] Jaime L., Santoyo S. (2021). The Health Benefits of the Bioactive Compounds in Foods. Foods.

[143] Vitaglione P., Donnarumma G., Napolitano A., Galvano F., Gallo A., Scalfi L., Fogliano V. (2007). Protocatechuic Acid Is the Major Human Metabolite of Cyanidin-Glucosides. J. Nutr..

[144] Matsumoto H., Inaba H., Kishi M., Tominaga S., Hirayama M., Tsuda T. (2001). Orally Administered Delphinidin 3-Rutinoside and Cyanidin 3-Rutinoside Are Directly Absorbed in Rats and Humans and Appear in the Blood as the Intact Forms. J. Agric. Food Chem..

[145] Gu J., Thomas-Ahner J., Riedl K., Bailey M., Vodovotz Y., Schwartz S.J., Clinton S.K. (2019). Dietary Black Raspberries Impact the Colonic Microbiome and Phytochemical Metabolites in Mice. Mol. Nutr. Food Res..

[146] Borges G., Roowi S., Rouanet J.-M., Duthie G.G., Lean M.E.J., Crozier A. (2007). The bioavailability of raspberry anthocyanins and ellagitannins in rats. Mol. Nutr. Food Res..

[147] Baron G., Altomare A., Regazzoni L., Redaelli V., Grandi S., Riva A., Morazzoni P., Mazzolari A., Carini M., Vistoli G. (2017). Pharmacokinetic profile of bilberry anthocyanins in rats and the role of glucose transporters: LC–MS/MS and computational studies. J. Pharm. Biomed. Anal..

[148] Toydemir G., Boyacioglu D., Capanoglu E., van der Meer I.M., Tomassen M.M.M., Hall R.D., Mes J.J., Beekwilder J. (2013). Investigating the Transport Dynamics of Anthocyanins from Unprocessed Fruit and Processed Fruit Juice from Sour Cherry (*Prunus cerasus* L.) across Intestinal Epithelial Cells. J. Agric. Food Chem..

[149] Kosińska-Cagnazzo A., Diering S., Prim D., Andlauer W. (2015). Identification of bioaccessible and uptaken phenolic compounds from strawberry fruits in in vitro digestion/Caco-2 absorption model. Food Chem..

[150] Akbari-Alavijeh S., Shaddel R., Jafari S.M. (2020). Encapsulation of food bioactives and nutraceuticals by various chitosan-based nanocarriers. Food Hydrocoll..

[151] Sharif N., Khoshnoudi-Nia S., Jafari S.M. (2020). Nano/microencapsulation of anthocyanins; a systematic review and meta-analysis. Food Res. Int..

[152] Baliyan N., Rani R., Kaur P., Yadava Y.K., Kumar L. (2020). Nanoencapsulation Development for Interactive Foods. Chem Sci Rev Lett.

[153] Yao L., Xu J., Zhang L., Liu L. (2021). Nanoencapsulation of anthocyanin by an amphiphilic peptide for stability enhancement. Food Hydrocoll..

[154] Tan C., Huang M., Wang J., Sun B. (2021). Biopolyelectrolyte complex (bioPEC)-based carriers for anthocyanin delivery. Food Hydrocoll. Health.

[155] Ribeiro M., Estevinho B.N., Rocha F. (2020). Microencapsulation of polyphenols—The specific case of the microencapsulation of *Sambucus Nigra* L. extracts—A review. Trends Food Sci. Technol..

[156] Tie S., Tan M. (2022). Current Advances in Multifunctional Nanocarriers Based on Marine Polysaccharides for Colon Delivery of Food Polyphenols. J. Agric. Food Chem..

[157] Bordenave N., Hamaker B.R., Ferruzzi M.G. (2014). Nature and consequences of non-covalent interactions between flavonoids and macronutrients in foods. Food Funct..

[158] Bao C., Jiang P., Chai J., Jiang Y., Li D., Bao W., Liu B., Liu B., Norde W., Li Y. (2019). The delivery of sensitive food bioactive ingredients: Absorption mechanisms, influencing factors, encapsulation techniques and evaluation models. Food Res. Int..

[159] Morris G.A., Kök S.M., Harding S.E., Adams G.G. (2010). Polysaccharide drug delivery systems based on pectin and chitosan. Biotechnol. Genet. Eng. Rev..

[160] Santiago L.G., Castro G.R. (2016). Novel technologies for the encapsulation of bioactive food compounds. Curr. Opin. Food Sci..

[161] Sreerekha P., Dara P.K., Vijayan D.K., Chatterjee N.S., Raghavankutty M., Mathew S., Ravishankar C.N., Anandan R. (2021). Dietary supplementation of encapsulated anthocyanin loaded-chitosan nanoparticles attenuates hyperlipidemic aberrations in male Wistar rats. Carbohydr. Polym. Technol. Appl..

[162] Jhaveri J., Raichura Z., Khan T., Momin M., Omri A. (2021). Chitosan Nanoparticles-Insight into Properties, Functionalization and Applications in Drug Delivery and Theranostics. Molecules.

[163] Fathi M., Martín Á., McClements D.J. (2014). Nanoencapsulation of food ingredients using carbohydrate based delivery systems. Trends Food Sci. Technol..

[164] Rosales TK O., da Silva M.P., Lourenço F.R., Hassimotto NM A., Fabi J.P. (2021). Nanoencapsulation of anthocyanins from blackberry (*Rubus* spp.) through pectin and lysozyme self-assembling. Food Hydrocoll..

[165] Koh J., Xu Z., Wicker L. (2020). Binding kinetics of blueberry pectin-anthocyanins and stabilization by non-covalent interactions. Food Hydrocoll..

[166] Arroyo-Maya I.J., McClements D.J. (2015). Biopolymer nanoparticles as potential delivery systems for anthocyanins: Fabrication and properties. Food Res. Int..

[167] Fang J.-L., Luo Y., Yuan K., Guo Y., Jin S.-H. (2019). Preparation and evaluation of an encapsulated anthocyanin complex for enhancing the stability of anthocyanin. LWT.

[168] Ge J., Yue X., Wang S., Chi J., Liang J., Sun Y., Gao X., Yue P. (2019). Nanocomplexes composed of chitosan derivatives and β-Lactoglobulin as a carrier for anthocyanins: Preparation, stability and bioavailability in vitro. Food Res. Int..

[169] Ge J., Yue P., Chi J., Liang J., Gao X. (2018). Formation and stability of anthocyanins-loaded nanocomplexes prepared with chitosan hydrochloride and carboxymethyl chitosan. Food Hydrocoll..

[170] Bulatao R.M., Samin J.P.A., Salazar J.R., Monserate J.J. (2017). Encapsulation of Anthocyanins from Black Rice (*Oryza sativa* L.) Bran Extract using Chitosan-Alginate Nanoparticles. J. Food Res..

[171] He B., Ge J., Yue P., Yue X., Fu R., Liang J., Gao X. (2017). Loading of anthocyanins on chitosan nanoparticles influences anthocyanin degradation in gastrointestinal fluids and stability in a beverage. Food Chem..

[172] Zhao X., Zhang X., Tie S., Hou S., Wang H., Song Y., Rai R., Tan M. (2020). Facile synthesis of nano-nanocarriers from chitosan and pectin with improved stability and biocompatibility for anthocyanins delivery: An in vitro and in vivo study. Food Hydrocoll..

[173] De Queiroz T., DupeyrAAn D., Carvalho J., GaivAAo I., Maistro E.L. (2018). Anthocyanins-loaded Eudragit® L100 nanoparticles: In vitro cytotoxic and genotoxic analysis. Genet. Mol. Res..

[174] Fernandes A., Oliveira J., Fonseca F., Silva F., Mateus N., Vincken J.-P., Freitas V. (2020). Molecular binding between anthocyanins and pectic polysaccharides—Unveiling the role of pectic polysaccharides structure. Food Hydrocoll..

[175] Chen M.-C., Mi F.-L., Liao Z.-X., Hsiao C.-W., Sonaje K., Chung M.-F., Hsu L.-W., Sung H.-W. (2013). Recent advances in chitosan-based nanoparticles for oral delivery of macromolecules. Adv. Drug Deliv. Rev..

[176] Mudgil D., Barak S. (2013). Composition, properties and health benefits of indigestible carbohydrate polymers as dietary fiber: A review. Int. J. Biol. Macromol..

[177] Noreen A., Nazli Z.i.H., Akram J., Rasul I., Mansha A., Yaqoob N., Iqbal R., Tabasum S., Zuber M., Zia K.M. (2017). Pectins functionalized biomaterials; a new viable approach for biomedical applications: A review. Int. J. Biol. Macromol..

[178] Luo Y., Wang Q. (2014). Recent development of chitosan-based polyelectrolyte complexes with natural polysaccharides for drug delivery. Int. J. Biol. Macromol..

[179] Jalili-Firoozinezhad S., Filippi M., Mohabatpour F., Letourneur D., Scherberich A. (2020). Chicken egg white: Hatching of a new old biomaterial. Mater. Today.

[180] Sui X., Sun H., Qi B., Zhang M., Li Y., Jiang L. (2018). Functional and conformational changes to soy proteins accompanying anthocyanins: Focus on covalent and non-covalent interactions. Food Chem..

[181] Zang Z., Chou S., Si X., Cui H., Tan H., Ding Y., Liu Z., Wang H., Lang Y., Tang S. (2021). Effect of bovine serum albumin on the stability and antioxidant activity of blueberry anthocyanins during processing and in vitro simulated digestion. Food Chem..

[182] Fathi M., Mozafari M., Mohebbi M. (2012). Nanoencapsulation of food ingredients using lipid based delivery systems. Trends Food Sci. Technol..

[183] Nguyen S., Alund S.J., Hiorth M., Kjøniksen A.-L., Smistad G. (2011). Studies on pectin coating of liposomes for drug delivery. Colloids Surf. B Biointerfaces.

[184] Fernández E.J., Ruyra A., Roher N., Zuasti E., Infante C., Fernandez-Diaz C. (2014). Nanoparticles as a novel delivery system for vitamin C administration in aquaculture. Aquaculture.

[185] Bamba B.S.B., Shi J., Tranchant C.C., Xue S.J., Forney C.F., Lim L.-T., Xu W., Xu G. (2018). Coencapsulation of Polyphenols and Anthocyanins from Blueberry Pomace by Double Emulsion Stabilized by Whey Proteins: Effect of Homogenization Parameters. Molecules.

[186] Chi J., Ge J., Yue X., Liang J., Sun Y., Gao X., Yue P. (2019). Preparation of nanoliposomal carriers to improve the stability of anthocyanins. LWT.

[187] Sekhon B.S. (2010). Food nanotechnology—An overview. Nanotechnol. Sci. Appl..

[188] Manzoor M., Singh J., Bandral J.D., Gani A., Shams R. (2020). Food hydrocolloids: Functional, nutraceutical and novel applications for delivery of bioactive compounds. Int. J. Biol. Macromol..

[189] Singh M., Hemant K., Ram M., Shivakumar H. (2010). Microencapsulation: A promising technique for controlled drug delivery. Res. Pharm. Sci..

[190] Akhavan S., Mahdi S., Assadpour E. (2016). Storage stability of encapsulated barberry’s anthocyanin and its application in jelly formulation. J. Food Eng..

[191] Dhakane J.P., Kar A., Patel A.S., Khan I. (2017). Effect of soy proteins and emulsification- evaporation process on physical stability of lycopene emulsions. Int. J. Chem. Studies.

[192] Patel A.S., Lakshmibalasubramaniam S., Nayak B. (2020). Steric stabilization of phycobiliprotein loaded liposome through polyethylene glycol adsorbed cellulose nanocrystals and their impact on the gastrointestinal tract. Food Hydrocoll..

[193] Mohammadalinejhad S., Kurek M. (2021). Microencapsulation of Anthocyanins—Critical Review of Techniques and Wall Materials. Appl. Sci..

[194] Pieczykolan E., Kurek M.A. (2019). Use of guar gum, gum arabic, pectin, beta-glucan and inulin for microencapsulation of anthocyanins from chokeberry. Int. J. Biol. Macromol..

[195] Shishir M.R.I., Xie L., Sun C., Zheng X., Chen W. (2018). Advances in micro and nano-encapsulation of bioactive compounds using biopolymer and lipid-based transporters. Trends Food Sci. Technol..

[196] Dini C., Islan G.A., Castro G.R. (2014). Characterization and Stability Analysis of Biopolymeric Matrices Designed for Phage-Controlled Release. Appl. Biochem. Biotechnol..

[197] Liao M., Ma L., Miao S., Hu X., Liao X., Chen F., Ji J. (2021). The in-vitro digestion behaviors of milk proteins acting as wall materials in spray-dried microparticles: Effects on the release of loaded blueberry anthocyanins. Food Hydrocoll..

[198] Tarone A.G., Cazarin C.B.B., Junior M.R.M. (2020). Anthocyanins: New techniques and challenges in microencapsulation. Food Res. Int..

[199] Ravanfar R., Tamaddon A.M., Niakousari M., Moein M.R. (2016). Preservation of anthocyanins in solid lipid nanoparticles: Optimization of a microemulsion dilution method using the Placket–Burman and Box–Behnken designs. Food Chem..

[200] Liang T., Zhang Z., Jing P. (2019). Black rice anthocyanins embedded in self-assembled chitosan/chondroitin sulfate nanoparticles enhance apoptosis in HCT-116 cells. Food Chem..

[201] Chatterjee N.S., Dara P.K., Raman S.P., Vijayan D.K., Sadasivam J., Mathew S., Ravishankar C.N., Anandan R. (2021). Nanoencapsulation in low-molecular-weight chitosan improves in vivo antioxidant potential of black carrot anthocyanin. J. Sci. Food Agric..

[202] Cui H., Si X., Tian J., Lang Y., Gao N., Tan H., Bian Y., Zang Z., Jiang Q., Bao Y. (2021). Anthocyanins-loaded nanocomplexes comprising casein and carboxymethyl cellulose: Stability, antioxidant capacity, and bioaccessibility. Food Hydrocoll..

